# Heritability of motor control and motor learning

**DOI:** 10.1002/phy2.188

**Published:** 2013-12-17

**Authors:** Julia Missitzi, Reinhard Gentner, Angelica Misitzi, Nickos Geladas, Panagiotis Politis, Vassilis Klissouras, Joseph Classen

**Affiliations:** 1 Ergophysiology Research Laboratory, Department of Sport Medicine and Biology of Physical Activity, University of Athens, Athens, Greece; 2 Human Cortical Physiology and Motor Control Laboratory, Department of Neurology, University of Wurzburg, Wurzburg, Germany; 3 School of Medicine, University of Crete, Heraklion, Greece; 4 Histology Laboratory, Center of Basic Research, Biomedical Research Foundation, Academy of Athens, Athens, Greece (P.P.); 5 Human Motor Control and Neuroplasticity Laboratory, Department of Neurology, University of Leipzig, Leipzig, D‐04103, Germany

**Keywords:** BDNF, dynamic motor training, force control, genetic variation

## Abstract

The aim of this study was to elucidate the relative contribution of genes and environment on individual differences in motor control and acquisition of a force control task, in view of recent association studies showing that several candidate polymorphisms may have an effect on them. Forty‐four healthy female twins performed brisk isometric abductions with their right thumb. Force was recorded by a transducer and fed back to the subject on a computer screen. The task was to place the tracing of the peak force in a force window defined between 30% and 40% of the subject's maximum force, as determined beforehand. The initial level of proficiency was defined as the number of attempts reaching the force window criterion within the first 100 trials. The difference between the number of successful trials within the last and the first 100 trials was taken as a measure of motor learning. For motor control, defined by the initial level of proficiency, the intrapair differences in monozygotic (MZ) and dizygotic (DZ) twins were 6.8 ± 7.8 and 13.8 ± 8.4, and the intrapair correlations 0.77 and 0.39, respectively. Heritability was estimated at 0.68. Likewise for motor learning intrapair differences in the increment of the number of successful trials in MZ and DZ twins were 5.4 ± 5.2 and 12.8 ± 7, and the intrapair correlations 0.58 and 0.19. Heritability reached 0.70. The present findings suggest that heredity accounts for a major part of existing differences in motor control and motor learning, but uncertainty remains which gene polymorphisms may be responsible.

## Abstract (precis)

Individual differences in motor control and learning are attributed to a great extent to genetic predisposition. However, uncertainty remains which gene polymorphism may be responsible.

## Introduction

Practice with feedback is a fundamental variable that influences motor skills. However, although everyone can improve with practice, some improve more than others. Moreover, people without previous experience perform certain activities better than others who have been practicing for years. Even in groups showing similar attainment, retrospective studies show individual differences in accumulated practise (Starkes et al. 1996). These differences in skill might arise from among other factors, different degrees of proximity of initial performance to the target performance, different conformity to optimal training, or gene‐mediated differences in responses to training (Yarrow et al. 2009).

Until recently, the question of the relative importance of genetic and environmental influences on motor control and motor learning was open, as previous studies have been confounded by a number of other biological and behavioral factors (Sklad 1972; Williams and Gross 1980; Fox et al. 1996). However, recently behavioral evidence was found that the brain derived neurotrophic factor (BDNF) val66met polymorphism may be a key factor influencing practice‐induced plasticity and motor learning (Kleim et al. 2006; Cirillo et al. 2012) suggesting a major genetic influence. Evidence obtained in both humans and animals confirmed this behavioral finding and has additionally supported the hypothesis that the same polymorphism also modulates the formation of long‐term potentiation (LTP), a major candidate mechanism of motor learning (Fritsch et al. 2010). Using a paired associative stimulation protocol (PAS) (Stefan et al. 2000), which likely probes LTP of excitatory synapses in motor cortex (Müller‐Dahlhaus et al. 2010), we found that variability in PAS‐induced plasticity was smaller between monozygotic (MZ) as compared to dizygotic (DZ) twins (Missitzi et al. 2011). We also showed that the susceptibility to PAS‐induced plasticity was significantly influenced by the BDNF val66met polymorphism (Missitzi et al. 2011) in agreement with previous work (Cheeran et al. 2008). Based on this previous work, we hypothesized that the same genetic variation that influences PAS‐induced plasticity could influence motor learning.

We undertook to assess the relative power of genetic and environmental contribution to the variation observed in motor control and learning using the classical twin method based on a comparison of MZ and DZ twins. Motor control was examined by the initial level of proficiency and motor learning by the improvement between the initial level of proficiency and the final level of attainment after dynamic training with feedback in force control.

## Methods

### Subjects

Forty‐four healthy female twins, (13 MZ and 9 DZ pairs, aged 24.6 ± 2.9 and 23.5 ± 3.2 years, respectively) were selected from a university student population to voluntarily participate in this study. With the addition of six more pairs, the population of subjects was identical to that studied in Missitzi et al. (2011). Special control was made for all confounding factors, as environmental comparability is a fundamental assumption made in the twin model. To ensure that environmental influences are comparable in both types of twins a questionnaire was administered regarding physical activity profiles, sport participation, occupational physical loading of the upper extremity, such as playing a musical instrument or using the computer (Baecke et al. 1982), socioeconomic status, and health condition (Table 1). None of the subjects had a history of serious medical, neurological or psychiatric illness, or used illegal, neuroactive recreational drugs as probed by a standardized questionnaire. All volunteers were right handed, except one twin pair, which was left handed according to the Oldfield handedness inventory (Oldfield 1971). The study was approved by University Institutional ethics committee and written informed consent was obtained from all participants. Zygosity was assessed through direct observation of relevant morphological characteristics, physical similarities, as well as the testimony of the obstetrical archives (Kasriel and Eaves 1976; Chen et al. 1999) and confirmed by serological examination of genetic markers in all twins. Discordance for a single antiserum was regarded as sufficient evidence of dizygosity (Sutton et al. 1962). BDNF genotyping was carried out twice in 14 subjects (four DZ and three MZ pairs) with a method described previously (Missitzi et al. 2011).

**Table 1.** Subjects demographics scores.

	MZ	DZ
Age (years)	22.9 ± 2.8	23.7 ± 3.7
Weight(kg)	56.3 ± 6.5	59.2 ± 10.3
Height (cm)	165.5 ± 4.8	167.1 ± 6.1
Instrument playing (h/week)	1.0 ± 0.5	1.5 ± 0.2
Keyboard writing (h/week)	3.9 ± 1.0	4.1 ± 2.3
Oldfield handedness score	0.9 ± 0.0	0.8 ± 0.0
Physical activity score	8.5 ± 2.1	8.3 ± 2.9

All *P* > 0.05.

### Experimental procedures

Subjects complied with pretest instructions that restricted alcohol and caffeine consumption during the 24 h prior to the tests and all were informed of the importance of having adequate sleep, during the night preceding the tests. To minimize possible circadian influences, experiments were started no more than 2 h apart in each pair, between 10:00 am and 4:00 pm.

**Figure 1.**
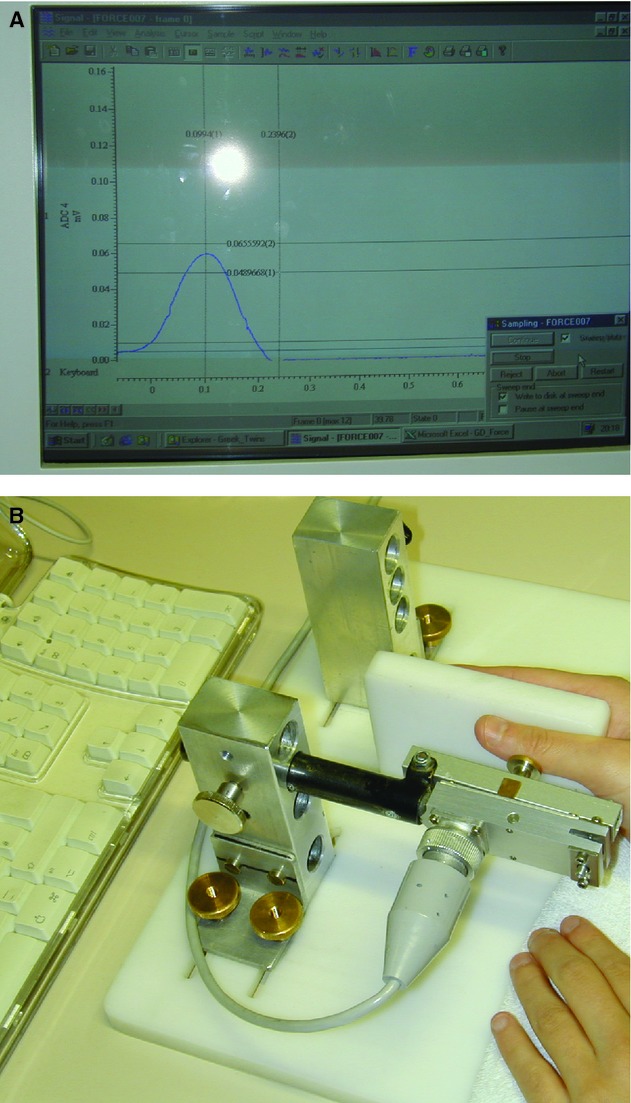
Test apparatus and the recording from one successive hit displayed between the two horizontal lines on the computer screen.

Experiments were performed in a quiet room with an ambient temperature of 21–22°C. Subjects were asked to perform brisk isometric abductions with their right thumb. Force was recorded by a force transducer (Grass CP122A, Grass Instruments CO, West Warwick, RI) and the force signal was fed back to the subject on a computer screen. Prior to the main task, the subject's maximum force was established, and a target force window was defined as a range between 30% and 40%, of the individual maximum force, displayed as two horizontal lines on the computer screen. Because in each experiment the display was scaled to the subject's individual maximum force, the target window had the same geometrical size for all subjects (Fig. 1).

#### Assessment of motor control

Motor control was defined as the initial level of proficiency. Each subject performed two blocks consisting of 50 isometric thumb abductions each, separated by 30 sec, at a frequency of 0.5 Hz. The total number of successful attempts achieved in the two blocks was used to assess motor control.

#### Assessment of motor learning

Motor learning was defined by the difference between the initial and last level of proficiency and was assessed by the difference between the total number of successful hits achieved in the last two training blocks, and the total number of successful hits achieved in the first two training blocks. Each subject had to perform a total of 500 metronome‐paced (0.5 Hz) isometric thumb abductions, exactly at the target location on the screen, in a series of 10 training blocks that were separated by 60 sec and consisted of 50 abductions each.

### Heritability estimates

Heritability (*h*^2^) which is defined as the proportion of phenotypic variance attributable to observed individual differences in actualized genetic potential was estimated on the basis of the intrapair difference between MZ and DZ twins. MZ twins have identical heredity, whereas DZ twins, like ordinary siblings, share half of their segregating genes. In this way, it is possible to separate the relative contribution of genotype and environment for the observed differences in motor control and learning (Klissouras et al. 2007). Data obtained were analyzed using the single‐factor analysis of variance (ANOVA) for each variable, to determine the significance of the differences, between the mean MZ and DZ intrapair variance, taking into consideration genetic type and pair factor. The variance ratio (*F*) derived from the single‐factor ANOVA determined whether further analysis was necessary. The following Clark equation based on intrapair variance was used to estimate heritability: *h*^2^* = *(*s*^2^**DZ* − s*^2^**MZ/*s*^*2*^**DZ)* × *100, where *s*^2^ DZ is the variance of intrapair differences in DZ twins and *s*^2^ MZ is the variance of intrapair differences in MZ twins. The computation of *h*^2^ was carried out, provided that the difference in genetic variance (within groups mean square) between the twin types (*F*‐test) was significant and the difference between means (*t*′‐test) and total variance (within plus between groups mean square) of both types of twins (*F′*‐test), which shows the homogeneity of the sample, was nonsignificant (Christian 1979). It is assured, therefore, that parameters assessed are independent from the twin type.

Statistical analyses were performed using SPSS (12.0 for Windows, SPSS Inc., Chicago, IL) and statistical functions built in Excel 2002 (Microsoft Corporation, Redmond, WA). Since our total sample size was *n* = 44 it follows that with type I error probability at 0.05, and the smallest expected difference between MZ and DZ set at *h*² = 0.50, a power level of at least at 95% is secured in this analysis (Dixon and Massiu 1985).

## Results

### Characteristics of the subjects

MZ and DZ twins did not differ in any of the demographic variables (Table 1) Furthermore, physical activity as assessed with a physical activity score (Baecke et al. 1982) was also similar within pairs, as well as between zygosity groups (Table 1).

**Figure 2.**
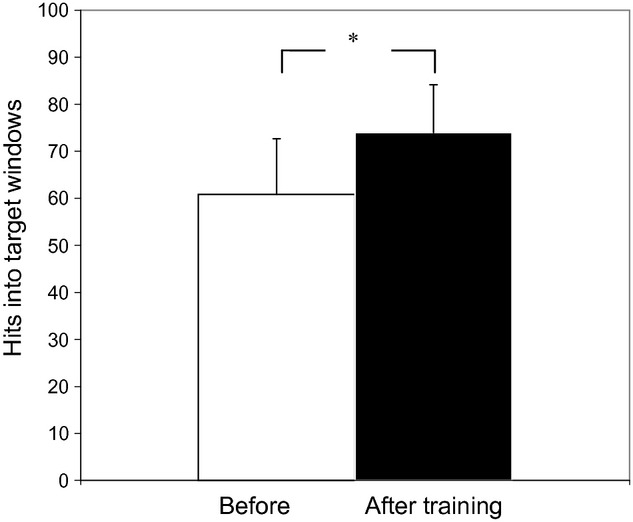
Mean and standard deviation in hits into target windows before (100 trials in two blocks) and after training (100 trials in two blocks). Asterisk indicates significant difference (paired *t* test; *P = 0.05).

Taking all experiments into consideration, initial performance assessed by the two first series was 60.8 ± 11.2. During training the force trajectories gradually became smoother and the number of hits into the force target zone increased. The outcome of training assessed by the two last series was 73.8 ± 10 (23%, *P *<**0.001, Fig. 2). The number of hits increased similarly in groups MZ (from 60.4 ± 9.4 to 72.2 ± 9.4, *P *<**0.001, paired two‐tailed *t*‐test) and DZ (61.8 ± 12.6 to 76.8 ± 10.6, *P < *0.001).

### Heritability of motor control

**Figure 3.**
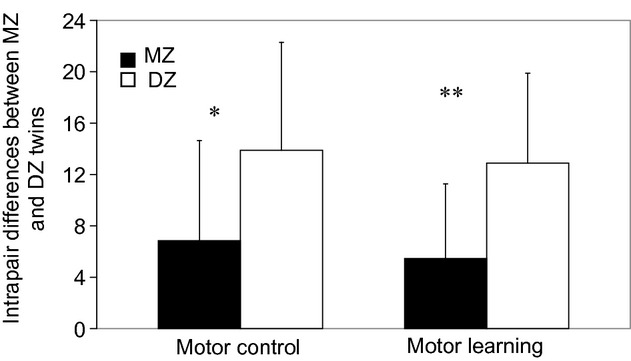
Mean and standard deviation of intrapair differences between MZ and DZ twins in motor control and motor learning. Asterisks indicate significant differences (paired *t* test; *P = 0.05 and *P = 0.01, respectively).

**Figure 4.**
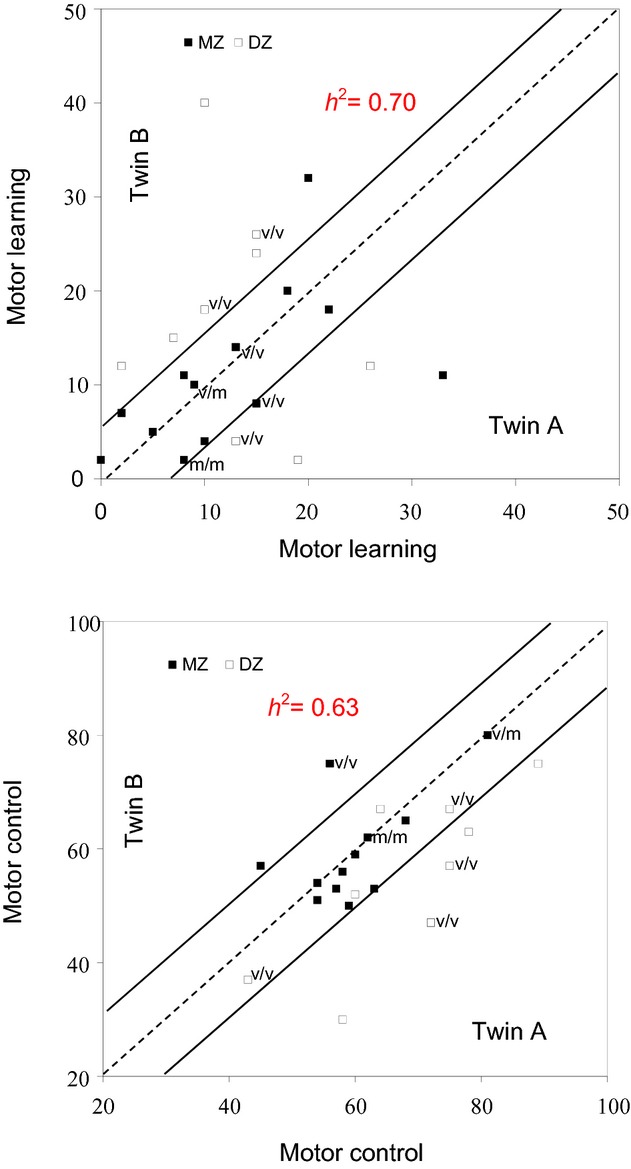
Individual values of motor learning and motor control in MZ and DZ twin pairs. BDNF allelic state is also indicated for some twin pairs, of whom three DZ and two MZ pairs are Val/Val carriers (v/v), one MZ pair Val/Met (v/m) and one MZ pair Met/Met (m/m).

Motor control was defined by the initial level of proficiency. The number of successful attempts of the first two blocks of exercise ranged from 45 to 80 hits into target windows for MZ and 43–75 for DZ twins. For most MZ twins’ performance were almost identical, whereas for DZ twins there were marked differences. Figure 3 presents means and standard deviations of intrapair differences in motor control as defined by the initial level of proficiency. Average intrapair differences between DZ twins were 13.8 ± 8.4 and between MZ 6.8 ± 7.8. The difference becomes more apparent in Figure 4, where the intrapair values for MZ are closer, and for DZ twins are more scattered. The respective intrapair correlation for MZ and DZ twins was 0.77 and 0.39. Statistical analysis of the data revealed that the differences between means and total variance of both types of twins were not significant, whereas the genetic variance between the twin types was significant *P < *0.05. Therefore, computation of *h*² was carried out and revealed that genetic factors explained 68% of the total variance.

### Heritability of motor learning

Motor learning was defined by the difference between the initial and last level of proficiency. Data obtained from the difference between the total number of successful hits achieved in the last two training blocks, and the total number of successful hits achieved in the first two training blocks were averaged for all MZ and DZ twin pairs. The results ranged from increments of 2–33 hits into target windows for MZ and 2–40 for DZ twins. A correlation was found (0.54) between the increment of force control and the baseline motor capacity. The correlation was similar in MZ as in DZ twins. Therefore, dependence on the initial level of proficiency is unlikely to explain the effect of zygosity.

In a subgroup of the present cohort, PAS‐induced plasticity was assessed (reported in Missitzi et al. 2011). Using Pearson's correlation coefficient we examined a potential relationship between the increment of force control and the baseline normalized magnitude of corticospinal excitability following PAS and we found a small but significant correlation (*r* = 0,21, *t* = 0.73 > tc = 0,66 *P *<**0.01).

Motor learning showed a greater intrapair similarity for MZ twins than for DZ twins. Intrapair differences between the two types of twins were more than double in DZ twins (12.8 ± 7) compared to MZ twins (5.4 ± 5.2; *P *<**0.01, Fig. 3). The difference becomes more apparent in Figure 4, where it can be seen that values of MZ twins are closer to the line of identity than values of DZ twins. The respective intrapair correlation for MZ and DZ twins was 0.58 and 0.19. Statistical analysis of the data revealed that the differences between means and total variance of both types of twins were not significant, whereas the genetic variance between the twin types was significant beyond the 0.01 level of confidence. Therefore, computation of *h*² was carried out and it was found that genetic factors explained 70% of the total variance.

### Genotyping results

Recent study has found that LTP formation and motor learning are both affected by the BDNF val66met polymorphism (Fritsch et al. 2010). By definition, MZ twins share the polymorphism of BDNF while this is not necessarily the case for DZ twins. Hence, it is possible that the closer intrapair differences found for MZ twins may be in a part due to the same BDNF haplotype, whereas the wider intrapair differences in DZ twins may have been due to a different BDNF haplotype. We were able to ascertain the BDNF haplotype in 14 twin pairs. As reported elsewhere (Missitzi et al. 2011), using the method applied by Neves‐Pereira et al. (2002), it was found that from this group of 14 twins, 10 were Val/Val carriers (3 DZ and 2 MZ pairs), 2 Val/Met (1 MZ pair) and 2 Met/Met (1 MZ pair). Sisters of the same pair had always the same haplotype in all pairs with known BDNF allelic state, even in heterozygous twins Regarding force control, subjects who carried Met in one or more alleles (*N* = 4) reached 71.2 ± 10.6 hits into target windows and did not show any significant difference from those having Val/Val who had outcomes of 63.4 ± 13.4 hits into target. With respect to learning, subjects who carried Met in one or more alleles (*N* = 4) improved by 9% (7 ± 3.4 hits; *P *<**0.01) after training with feedback. In contrast, those having Val/Val alleles (*N* = 10) improved on average by 20% (12.2 ± 6.6 hits, *P *<**0.001 Fig. 4).

## Discussion

Τhe current study demonstrated that existing interindividual differences on both force control and motor learning are under genetic influence, with heritability being 0.68 and 0.70, respectively.

### Motor control

Previous studies have reported a significant genetic effect for motor control, using a variety of tasks, such as pursuit rotor tracking, tapping speed, and stabilometry with heritability ranging from 0.56 to 0.86, depending on the task (Williams and Gross 1980; Fox et al. 1996; Maes et al. 1996). Nevertheless, by performing these tasks, the involvement of other biological and behavioral factors, like balance, power, proprioception, rhythm, perception, and motor learning, which may influence motor control, is inevitable, making its isolation difficult. An attempt was made, however, to examine neuromuscular coordination by kinematic and electromyographic recordings during a simple, single joint movement in one degree of freedom. A comparison of intrapair differences between MZ and DZ twins in neuromuscular coordination of fast movements, expressed either as movement accuracy or movement economy, demonstrated that heredity accounts for the major part (87%) of existing differences (Missitzi et al. 2004). The heritability estimate of motor control found in this study (0.68) was lower than that found previously relating to neuromuscular coordination measures, an observation that may be explained by the fact that different tasks challenge multiple sensory and motor capacities differently and are each subject to different heritability (J. Missitzi, A. Misitzi, N. Geladas, J. Classen, and V. Klissouras, unpubl. data).

Motor control was examined in the current study by performing brisk isometric abductions with the thumb, a movement of a single joint, in one degree of freedom in a simple protocol, used previously (Stefan et al. 2006). Simplicity of the motor task enhances the chances for thorough control of experimental factors and may thus minimize the influence of confounding factors (Corcos et al. 1989; Almeida et al. 1995). Despite the apparent simplicity of the motor task, it challenges the orchestration of multiple sources of sensory information, exteroceptive (vision) as well as proprioceptive, namely, the tendon organs’ sensitivity, the discharge of the major part of all muscle spindle afferents and the excitement of cutaneous receptors, through ensemble coding mechanisms, but without knowing the exact contribution of each (Edin and Valbo 1990; Jones and Piateski 2006) within the motor output system. Recently, it has been shown that isometric muscle contractions can produce a perception of joint displacement in the same direction as the joint would move if unrestrained (Walsh et al. 2009). In addition, hemiparetic participants seem to rely primarily on sense of effort rather than proprioceptive feedback for gauging lower limb force production for both isometric and isotonic contractions (Simon et al. 2009). Therefore, in this study along with afferent information, centrally generated motor command signals, which have been found to have a genetic basis (Missitzi et al. 2004), is likely to play a major role. Hence, any part of the system concerned with the generation of the force production task, collection or processing of afferent information or the generation of efferent signals may be genetically influenced.

It has been shown that BDNF val66met polymorphism is associated with reduced hippocampus volume and function, episodic and working memory, less gray matter volume throughout the prefrontal cortex (Egan et al. 2003; Hariri et al. 2003; Pezawas et al. 2004; Dempster et al. 2005; Tan et al. 2005; Ho et al. 2006). As the task used in this study probed motor, attentional, memory, and visuospatial systems, one might hypothesize BDNF polymorphism to play a role in the intrapair differences in motor control found between MZ and DZ twins. However, the mean number of successful attempts achieved from subjects who carried Met in one or more alleles (*N* = 4) did not show any significant difference from those having Val/Val (*N* = 10). Therefore, the wider intrapair differences in DZ twins in the motor control task employed here are unlikely to be due to a different haplotype of BDNF polymorphism. Alternatively, the BDNF polymorphism may play a role of motor skill acquisition in the short term (see below), but less so in the long term. On this view carriers of the BDNF gene Met allele would be at a disadvantage in the rapidity of motor skill acquisition, but able to compensate for this disadvantage, perhaps by other genes or by an advantageous effect of the Met allele in later stages of motor skill encoding (McHughen et al. 2011), such as in consolidation. However, it should be noted that on one hand the sample for detecting genotyping was small and further investigation is needed to exclude this possibility and on the other hand polymorphisms of other genes whose product is involved in motor control such as DRD2/ANKK1 Ankyrin repeat and kinase domain 1 (Munafo et al. 2005) or GCH1 GTP cyclohydrolase 1 (Tegeder et al. 2006), or GLRA1 Glycine receptor 1 (Elmslie et al. 1996) or other (Mishra et al. 2007) could be more relevant.

Hence, as motor control presents a high heritability, efforts to find a causative gene are worthwhile to continue and determine which gene polymorphisms or a combination thereof may underline the difference between MZ and DZ twins.

### Motor learning

A significant genetic variance component (0.70) was also found for motor learning. Previous studies reported a similar heritability index for the initial level of motor learning which increased further with practice (Williams and Gross 1980; Fox et al. 1996). In this study, both groups of twins started with a similar level of performance without significant differences and both improved significantly over the 10 sessions of motor practice. However, as motor practice continued although everyone improved some improved more than others. It is of importance to note that the results from the first two series, which were determined as the initial performance as well as from the improvement after the practice differ a lot between the participants and that some twins presented particularly good performance from the start which was maintained and increased over the course of the training. In our subgroup of 14 subjects in whom we were able to ascertain the BDNF gene polymorphisms, we found as above‐mentioned four individuals who carried at least one Met allele. Training led to a significantly smaller increase in motor learning in these four subjects; on the contrary, the remaining subjects carrying the Val/Val allelic state responded with an almost double increment. In accordance with this, the results of a recent study showed that the BDNF val66met polymorphism impairs motor skill acquisition in humans and mice (Fritsch et al. 2010). Furthermore, in a recent functional magnetic resonance imaging (MRI) study, McHughen and associates (McHughen et al. 2010) examined a single‐nucleotide polymorphism of the human BDNF gene in relation to brain motor system function, short‐term plasticity, and short motor learning and found that Val/Met polymorphism subjects of BDNF genotype showed poorer short‐term learning and retention on motor behavior tests relative to Val/Val subjects. Furthermore, previous study gives an indirect support of these results, as a significant increase in the motor evoked potentials amplitudes in abductor pollicis brevis after PAS was found in Val/Val, but no increase in non‐Val/Val individuals exposed to plasticity inducing brain stimulation protocols; which is supposed to be under the same neural substrate as motor learning (Cheeran et al. 2008). The twin cohort of this study on motor control and motor learning includes a cohort of twins in whom the genetic influence on externally induced plasticity was studied. In the previous study, it was demonstrated that the change in corticospinal excitability after an intervention with PAS was genetically dependent in a substantial part 68% (Missitzi et al. 2011), almost the same as in the results found in the current study for motor learning (70%).

As these studies examine the heritability of early motor learning and heritability of human brain short‐term plasticity; two parameters which are thought to be supported by the same mechanisms (Asanuma and Pavlides 1997; Rioult‐Pedotti et al. 2000) specifically in early phases of human motor learning (Rosenkranz et al. 2007), through unmasking of preexisting intracortical connections and increasing the efficacy of existing synaptic connections by LTP‐like plasticity, we could speculate that the same genotype influences their genetic variation to a similar degree. This would be entirely consistent with the fact that both studies activated the same muscle and was conducted to almost the same sample of twins. In the previous study, PAS intervention activated both intracortical pathways that were also active with the voluntary activity in the current study, together with the median nerve of the abductor pollicis brevis. This supports theoretical models that have been proposed as the basis of motor learning, as well as that the same mechanisms support plasticity of motor cortex and motor learning.

Our conclusion that plasticity of motor cortex and motor learning are associated through shared genes is in line with a similar genetic influence on intelligence and change in cortical thickness (Brans et al. 2010) and with the dependence of learning and memory formation on the plasticity of neural circuits (Escobar et al. 2008).

In this survey, the sisters of the same pair of our subgroup who have been genotyped had always the same allelic state in all pairs, even in heterozygous twins (Fig. 4). Therefore, it is difficult to draw conclusions about the influence of the BDNF polymorphisms on the difference between MZ and DZ twins. However, based on the assumption that Met alleles would occur at the same frequency as in the cohort of 14 subjects, two DZ pairs could be heterozygous for BDNF gene alleles. Given the large difference in responsiveness toward the training protocol in non‐Met and Met carriers, it is conceivable that the BDNF polymorphism may have substantially contributed to the wider intrapair difference for DZ twins.

In addition, by comparing the present results related to the motor skill acquisition, with previous ones on externally induced LTP‐like plasticity (Missitzi et al. 2011) we found a correlation between motor learning and the PAS‐induced plasticity results. This finding appears to provide evidence that the two measures are related, although the weakness of the correlation between them suggests a rather indirect relationship or the presence of significant other factors modulating the relationship between them. Individuals carrying at least one Met allele of the BDNF polymorphism exhibited both reduced ability for motor learning (this study) and brain plasticity (Missitzi et al. 2011). These observations, along with recent evidence that BDNF val66met polymorphism may be a major factor influencing practise‐induced brain plasticity, motor learning as well as modulating the formation of LTP (Kleim et al. 2006; Fritsch et al. 2010; Cirillo et al. 2012) strengthen and enhance the possibility that BDNF gene polymorphism may influence both motor learning and neuronal plasticity and be partly responsible for the differences observed between the two types of twins. It has to be noted, however, that BDNF gene polymorphism is only one example of genetic susceptibility and that some controversy exists about the role of the BDNF polymorphism for motor learning (Li Voti et al. 2011; Freundlieb et al. 2012) while there is evidence for an interaction of the genes encoding BDNF and Catechol‐O‐methyltransferase (COMT) with respect to human cortical plasticity, and that genotype‐related differences in neurophysiology, translate into behavioral differences (Witte et al. 2012). Hence, more studies are needed to determine which gene polymorphisms and under which circumstances may underline the difference between MZ and DZ twins.

Recent evidence shows that after brief periods of movement training there are not only changes in motor function but also persistent changes to the way we perceive the position of our limbs (Feldman 2009; Ostry et al. 2010). Moreover, force field learning might in principle lead subjects to modify their estimates of limb position and to interpret somatosensory feedback during subsequent perceptual testing (Körding and Wolpert 2004). Therefore, the smallness of the correlation found between motor learning and PAS‐induced plasticity may indicate isolated motor adaptation in brain plasticity, as PAS does not require active involvement of the participant in the context of movement production, which is required for the sensory shift. Heritability indexes, however, for brain plasticity and motor learning were almost the same, regardless of the somatosensory system participation in motor learning, perhaps because individual variation in proprioception seems to be influenced also by genetic factors (J. Missitzi, A. Misitzi, N. Geladas, J. Classen, and V. Klissouras, unpubl. data).

Although recent studies have provided evidence that synaptic plasticity (Cheeran et al. 2008) as well as motor learning (McHughen et al. 2010) are genetically influenced, independent and complementary information could be gained from twin studies, especially in these multifactorial characteristics which are unlikely to be influenced by a single gene. Our findings elucidate the genetic effect on individual differences in force control and motor learning and support studies that integrate genomics with developmental biology, for understanding the molecular and genetic mechanisms that govern the limits of athletic performance. Adaptive changes are essential for the consolidation of a memory of performance and therefore for the lasting ability of performing highly skilled movements, like those required for Olympic performance (Nielsen and Cohen 2008) As the same external intervention does not induce the same adjustments and it does not lead to the same activation levels, either for learning a motor skill or master a task to perfection, it may be that athletes of Olympic caliber in addition to their superior genotypes may also have inherited to some degree the cortical ability to better respond to motor training. These results not only could provide an insight for performance variation in sports which require high phenotype in motor skills, and learning but also in professions which require movements executed with precision and economy, such as pilots, dancers, musicians, and surgeons.

The present findings may be relevant to understand why people express different adaptive central nervous system response patterns to various injuries. Functional deficiencies and recovery outcomes differ widely between patients with identical peripheral injuries (Kapreli et al. 2007) possibly as a result of different expressions of motor learning. The fact that heredity accounts for a substantial part of the existing differences in human motor learning capacity may also imply that this influence is a factor in the development of or compensation of certain neurological injuries. On the basis of the aforementioned studies and based on our findings, we consider it likely that the differences in rehabilitation after an injury as well as in any type of motor skill acquisition in sports, or in professional, artistic, and recreational activities may be in part genetically influenced.

## Acknowledgments

The authors thank George Vagenas for advice in statistical analysis, Vassilis Kouvelas for the zygosity determination, Thomas Stauss and Leonidas Misitzis for assistance in providing the sample of twins, as well as the twins for their enthusiastic participation in the study.

## Conflict of Interest

None declared.
